# Supplementary material for: Efficacy of Combination Therapy with Lenvatinib and Radioactive Iodine in Thyroid Cancer Preclinical Model
Source: Int J Mol Sci. 2022 Aug 30;23(17):9872. doi: 10.3390/ijms23179872 (PMC9456011; doi:10.3390/ijms23179872)
Supplement: Supplementary file 1 [file ijms-23-09872-s001.zip › ijms-1872128-supplementary.pdf]

Title

Efficacy of Combination Therapy with Lenvatinib and Radioactive Iodine in Thyroid Cancer Preclinical Model

Kensuke Suzuki [1,*], Hiroshi Iwai [1], Keita Utsunomiya [2], Yumiko Kono [2], Tadashi Watabe [3], Yoshiki Kobayashi [1], Dan Van Bui [1], Shunsuke Sawada [1], Yasutaka Yun [1], Akitoshi Mitani [1], Kenta Fukui [1], Haruka Sakai [1], Hanh Hong Chu [1], Nguyen Manh Linh [1], Noboru Tanigawa [2] and Akira Kanda [1]

Affiliations

1 Department of Otolaryngology, Head and Neck Surgery, Kansai Medical University, Hirakata 573-1010, Osaka, Japan

2 Department of Radiology, Kansai Medical University, Hirakata 573-1010, Osaka, Japan

3 Department of Nuclear Medicine and Tracer Kinetics, Graduate School of Medicine, Osaka University, Suita 565-0871, Osaka, Japan

* Correspondence: suzukken@hirakata.kmu.ac.jp; Tel.: +81-72-804-0101 (ext. 56490)

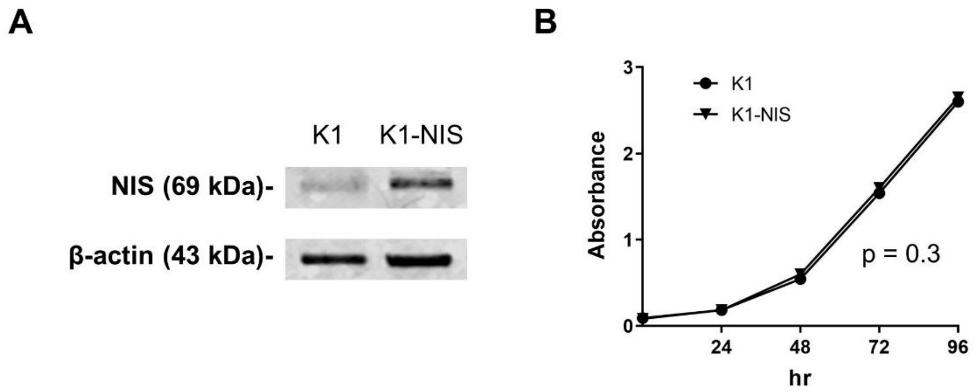

Supplementary Figure S1. Immunoblot and cell proliferation assay of thyroid cancer cell lines. (**A**) Sodium iodide symporter (NIS) protein expression in K1 and NIS stably transfected K1 cell lines (K1-NIS). Collected cellular proteins from K1 or K1-NIS cells were electrophoresed and transferred to a PVDF membrane. The membranes were incubated with NIS and β-actin antibodies. (**B**) Cell proliferation assay of K1 and K1-NIS cells. Cells (2×10^3 cells/well) were seeded and cultured in 96-well culture plates for 0, 24, 48, 72, or 96 h. WST-8 at 10 μl was added to the wells, the cells were incubated for 4 h at 5% CO_2 and 37ºC, and then absorbance was measured at a wavelength of 450 nm (n = 4). Data represent means ± SEM.

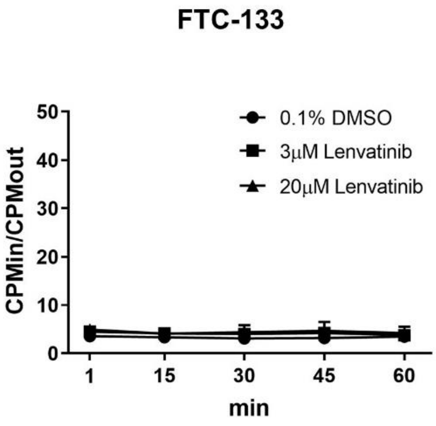

Supplementary Figure S2. Intracellular uptake of radioiodine after lenvatinib treatment *in vitro*. The intracellular accumulation ratio (CPMin/ CPMout) of ^{125}I in FTC-133 cells treated with 0.1% DMSO or 3 and 20 μM lenvatinib for 48 h. Cells at a concentration of 1–2 × 10^6 cells/mL were obtained at 1, 15, 30, 45, and 60 min after adding 370 kBq of ^{125}I, and the ratio of radioactivity concentration inside the cell to that found in the supernatant was calculated (n = 5). Data represent means ± SEM.
